# Dislocation dynamics modelling of the creep behaviour of particle-strengthened materials

**DOI:** 10.1098/rspa.2021.0083

**Published:** 2021-06

**Authors:** F. X. liu, A. C. F Cocks, E. Tarleton

**Affiliations:** ^1^ Department of Materials, University of Oxford, Parks Road, Oxford OX1 3PH, UK; ^2^ Department of Engineering Science, University of Oxford, Parks Road, Oxford OX1 3PJ, UK

**Keywords:** particle, self-climb, cross-slip, creep

## Abstract

Plastic deformation in crystalline materials occurs through dislocation slip and strengthening is achieved with obstacles that hinder the motion of dislocations. At relatively low temperatures, dislocations bypass the particles by Orowan looping, particle shearing, cross-slip or a combination of these mechanisms. At elevated temperatures, atomic diffusivity becomes appreciable, so that dislocations can bypass the particles by climb processes. Climb plays a crucial role in the long-term durability or creep resistance of many structural materials, particularly under extreme conditions of load, temperature and radiation. Here we systematically examine dislocation-particle interaction mechanisms. The analysis is based on three-dimensional discrete dislocation dynamics simulations incorporating impenetrable particles, elastic interactions, dislocation self-climb, cross-slip and glide. The core diffusion dominated dislocation self-climb process is modelled based on a variational principle for the evolution of microstructures, and is coupled with dislocation glide and cross-slip by an adaptive time-stepping scheme to bridge the time scale separation. The stress field caused by particles is implemented based on the particle–matrix mismatch. This model is helpful for understanding the fundamental particle bypass mechanisms and clarifying the effects of dislocation glide, climb and cross-slip on creep deformation.

## 1. Introduction

As a reliable, low-emitting and cost-competitive source of electricity, nuclear energy has become the second-largest source of low-carbon electricity production globally, which is playing an expanded role in creating a sustainable future energy system. In nuclear power plants, materials experience increasingly demanding environments of higher temperatures and radiation-induced damage, where permanent creep deformation occurs as a result of long-term exposure to stresses even below the material yield strength. This time-dependent creep deformation has long been a concern for engineers. Current time-dependent deformation is mainly evaluated based on empirical equations extracted from tests under standard conditions, with little connection to the underlying microstructural mechanisms, particularly for particle-strengthened alloys.

Microscopically, inelastic deformation occurs by the collective motion of crystal dislocations (line defects within a perfect crystal) [1]. These can glide on what are known as slip planes, leading to planes of atoms sliding over each other (glide motion), or climb out of the slip plane (climb motion) to overcome obstacles in their path. At low temperature (less than *T*_*m*_/3, where *T*_*m*_ is the absolute melting temperature), the deformation is dominated by dislocation glide. The rate of deformation due to dislocation glide is proportional to the mobile dislocation density and velocity of the dislocations (the Orowan equation) which is controlled by a variety of internal resistance forces, including the intrinsic resistance caused by the crystal lattice (the Peierls stress), the interaction with impurity (solute) atoms or particles and interactions with other dislocations. In a complete description, the strain rate depends on how quickly the dislocations overcome the barriers to their glide and how quickly they move from one barrier to the next, such that the strain rate can be adequately described in terms of the waiting time spent at barriers. While at elevated temperature, a dislocation that has been brought to a halt by an obstacle can overcome the obstacle and start moving again by a process called dislocation climb. For dislocation climb to occur, atomic diffusion has to be able to take place, either in the lattice, known as lattice diffusion, leading to a non-conservative climb motion, or along the dislocation core region, where atoms are loose-packed and highly disordered [2,3], allowing rapid matter transport even at lower temperatures. We refer to this latter process as core diffusion, and it results in a self-climb motion [4]. As with all diffusion processes, the climb motion is highly dependent on the temperature. At high temperatures, there is sufficient mobility of point defects or atoms to allow appreciable climb as well as glide motion, which facilitates the annihilation of dislocations [5] and the recovery of dislocation structures as the material creeps. Deformation is, therefore, possible at a much lower stress than would be required for glide alone. For this reason, many particle-hardened materials become significantly weaker at high temperatures [6,7]. Exploiting the hardening potential of particles in alloys then requires first understanding the interaction between dislocations and particles.

For particle strengthening, mobile dislocations are arrested or slowed down at the particles and extensive deformation can only occur when a threshold stress is exceeded. The dislocation can then escape from the pinning particles by one or a combination of the following bypass mechanisms: (1) particle cutting [8,9], either by fracture of incoherent particles or by the glide of dislocation pairs through coherent particles; (2) Orowan bowing between particles [10], where the dislocation is extended in the glide plane between particles by an applied stress until the dislocation is released, leaving shear loops around the particles; (3) cross-slip around the particles [11–13], where screw dislocation segments surmount a particle via the formation of prismatic dislocation loops; (4) climb over particles [7] where segments of the dislocation are extended normal to the original slip plane by diffusion of atoms towards or away from the climbing dislocations. Mechanisms (1)–(3) depend weakly on temperature, while mechanism (4) involves mass diffusion and thus is intrinsically slower than the others. Therefore, when either mechanism (1), (2) or (3) can occur it will dominate, particularly at high stresses and/or low temperatures. There is general agreement that at high temperatures and relatively lower stresses, when the athermal mechanisms are inhibited, both shearable or non-shearable particles are bypassed by climb.

Two extreme models for dislocation climb over individual particles have been proposed, the ‘local climb’ model and the ‘general climb’ model, where the threshold bypass stress is energetically determined according to the increase in dislocation line length as the dislocation bypasses a particle. The ‘local climb’ model, developed by Brown & Ham [14] and Shewfelt & Brown [15], postulates that the climbing dislocation segment profiles the particle, and the dislocation between the particles remains in its slip plane. As a significant amount of new dislocation line has to be created in the course of ‘local climb’, extra energy must be supplied, resulting in a relatively large threshold stress. Although this gives an appropriate order-of-magnitude estimate for creep thresholds measured in particle-strengthened materials, it can be argued [16] that ‘local climb’ represents an extremely unstable process; in practice, the sharp change in profile of the dislocation could be rapidly relaxed by diffusion, leading to a more ‘general climb’ profile. In the ‘general climb’ model, the dislocation gradually changes its profile between particles to minimize the curvature. This leads to an unrealistically small threshold stress [17]. It also requires much longer range diffusion than local climb and therefore resulting in a strain rate many orders of magnitude slower, especially for a low volume fraction of particles. Besides the energetics of the climb process itself, a detachment mechanism [18,19] is proposed at the particle–dislocation interface, in which an attractive particle–dislocation interaction is assumed at high temperature, to explain the experimental observations of dislocation pinning in crept oxide-dispersion strengthened alloys [20,21]. As demonstrated by Srolovitz *et al*. [18], an attractive interaction can result due to the dislocation at the interface relaxing part of its strain field. Arzt & Wilkinson [19] have further demonstrated that this attractive interaction provides a stronger barrier for dislocation bypass than the climb motion itself, thus increasing the stress required to bypass the particle. However, despite extensive studies of the particle bypass mechanisms where climb is significant, available models make a number of simplifying assumptions, for example, only the interaction between long straight dislocations and individual particles is considered [22]; dislocation climb is treated using a glide-like phenomenological mobility law [23], rather than as a physical diffusion-controlled process; or only lattice diffusion is accounted for in the climb motion, with core-diffusion controlled self-climb motion usually neglected. In fact, in a real alloy, interactions between a complicated dislocation network and a large number of particles is a rather complex process controlled by several competing mechanisms, which involve the coupling between glide and local climb [5] of edge segments and the cross-slip of screw segments. It is the collective microstructural behaviour that governs the deformation process at high temperature; systematic studies of which, to our knowledge, still remain scarce.

As in many physical problems involving the interactions of a large number of particles, the main difficulty in explaining the formation of complicated dislocation structures and the resulting deformation behaviour lies in understanding the collective dynamic behaviour of a large group of dislocations. A powerful approach, which can simultaneously retain key microstructural features of the dislocation network in terms of a manageable number of degrees of freedom, is provided by the numerical treatment of discrete dislocations. Over the years, extensive studies have been carried out to advance the state of knowledge and applicability of discrete dislocation dynamics (DDD) since it was proposed in the late 1980s [24], such as finite boundary value problems [25], the displacement field of a complex dislocation network [26], dislocation cross-slip [27], improvement in the computational efficiency in calculating the long-range stress and the numerical integrators [28,29], interaction with point-defects, particles or hydrogen atoms [30–32], creep and dislocation climb at elevated temperature [4,33–36]. However, microstructural evolution processes during the creep of particle-strengthened materials span a wide range of length and time scales, which exceed the capacity of the existing discrete dislocation (DD) method. Remaining challenges involve (i) how to bridge the huge time scale separation (greater than 10^6^) between dislocation slip and climb, to couple dislocation glide, climb and cross-slip in a unified framework; and (ii) extend the existing DD model to involve particles and point defects, to keep track of all the interactions between dislocations and various defects, to embrace the complex nature of real engineering alloys.

Recently, we have developed a new method which incorporates atomic diffusion into the nodal-based DD method and bridges the time scale separation between dislocation glide and self-climb [4,35]. This method acts as a steppingstone for the modelling of a new class of physical problems. With this new method and our GPU accelerated three-dimensional DDD model [29], the current work aims to extend the range of the current DD framework [4,35] from the ‘plasticity’ domain to the ‘creep’ domain, with emphasis on high-temperature dislocation behaviour in the presence of particles. The significance of undertaking this task is threefold: (i) developing a framework that captures the salient features of collective dislocation behaviour in the presence of particles; (ii) enabling a systematic analysis of the particle bypass mechanisms; and (iii) providing ‘bottom-up’ insights into the intrinsic mechanism of creep deformation.

The paper is organized as follows. We describe the methodology in §2 before presenting its validation of climb and cross-slip in §3. Then, results concerning creep processes during the compression of micropillars are discussed in §4, demonstrating the relevance of our model and bringing new insights. The conclusion is provided in §5.

## 2. Methodology

In this section, we develop a DD model that includes elastic interactions, dislocation glide, climb and cross-slip mechanisms, to provide a systematic interpretation of how dislocations interact with particles. In a conventional nodal-based DD framework, given the dislocation network, the stress field **σ**(*x*) can be computed on the basis of continuum linear elasticity theory. The stress field then produces driving forces ***F*** on the nodes, and the nodes respond to these forces by making discrete movements according to a specified mobility function ***M*** that is characteristic of the dislocation type and the specific material being simulated. Key inputs for a DD simulation, therefore, include (1) the driving force ***F*** on the dislocation segments, including the externally applied stress, dislocation line tension, the long-range dislocation interactions, and the short-range dislocation–particle interactions; (2) the dominated dislocation mobility laws ***M*** at specified loading conditions, temperatures and materials. In the current section, we develop precise expressions for the dislocation driving force ***F*** and physical mobility law ***M*** for particle-strengthened BCC materials at elevated temperature.

### (a) Driving force for dislocation motion

**Figure 1.**
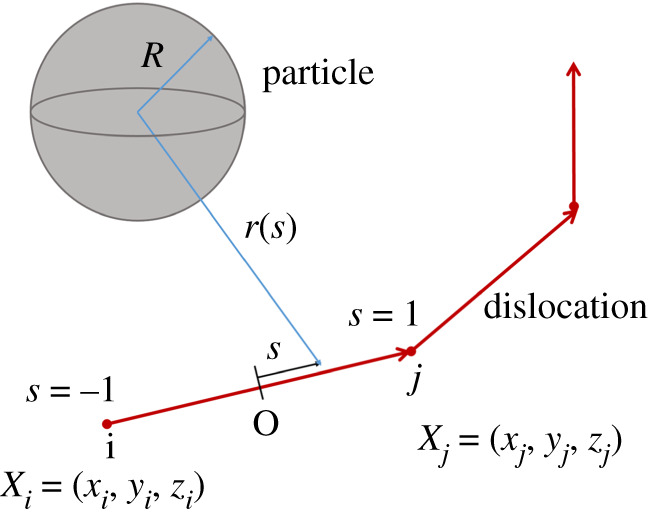
Schematic diagram of a system of interactions between a particle and dislocation segments. (Online version in colour.)

In the nodal-based DD framework, the non-singular continuum theory of dislocations is adopted [37]. Here, we follow the convention that the nodal values are denoted in uppercase and numbered by subscript. Consider a dislocation segment adjacent to a spherical particle of *R*, as shown in figure 1. The full force on a dislocation node *i*, ***F***_*i*_, in a particle-strengthened material, consists of five parts

2.1 $$F_{i}={\tilde{F}}_{i}+F_{i}^{\text{self}}+F_{i}^{\text{core}}+F_{i}^{\text{app}}+F_{i}^{pt},$$

where ${\tilde{F}}_{i}$ refers to elastic interaction between the *ij* segments connected to node *i* with every other segment *mn* (*mn* ≠ *ij*), $F_{i}^{\text{self}}$ denotes the elastic self force on node *i*, due to segments *ij* connected to node *i*, $F_{i}^{\text{core}}$ is the dislocation core force on node *i*, $F_{i}^{\text{app}}$ accounts for the externally applied stresses, and $F_{i}^{pt}$ is the nodal force caused by the particles. Details about ${\tilde{F}}_{i}$, $F_{i}^{\text{self}}$, $F_{i}^{\text{core}}$ and $F_{i}^{\text{app}}$ are provided in our previous work [4]. We, thus, limit our attention, in the current subsection, to implementing the continuum misfit stress field of the particles into the nodal-based three-dimensional DDD framework to calculate the driving force on the dislocation segments caused by the particles.

#### (i) Stress field of an impenetrable particle

Consider an impenetrable and non-deformable spherical particle, centred at C, with a radius of *R*, as shown in figure 1, made up of an elastic medium that is located in an infinite medium or matrix such that the radial misfit is *δ*. The misfit is described by the difference between the particle radius *R* and the matrix it replaces in the absence of the particle. As described by Eshelby [38,39], the stress in spherical coordinates generated in the matrix as a result of the misfit is given by

2.2 $$\sigma_{rr}=-\frac{4GM}{r^{3}}\;\text{and}\;\sigma_{\theta \theta }=\sigma_{\psi \psi }=\frac{2GM}{r^{3}}.$$

In the above equation, *M* = *δV*/4*π* ((1 + *ν*)/3(1 − *ν*)), where *δV* indicates the misfit volume between the particle and the hole, *ν* is Poisson’s ratio. For a small misfit, *δV* = 4*πR*^2^*δ* = 4*πR*^3^ε, with ε = *δ*/*R* denoting the dilatational misfit strain. *G* is the shear modulus, *r* is the distance from the field point (*r*, *θ*, *ψ*) to the centre of the particle *C*. Here, we assume the same elastic properties for the particle and the matrix, so that there are no image forces at the interface between the matrix and the particle.

Equation (2.2) can be rewritten as

2.3 $$\sigma_{rr}=-\frac{4G\varepsilon R^{3}}{r^{3}}\frac{1+\nu }{3(1-\nu )}\;\text{and}\;\sigma_{\theta \theta }=\sigma_{\psi \psi }=\frac{2G\varepsilon R^{3}}{r^{3}}\frac{1+\nu }{3(1-\nu )}.$$

As demonstrated in equation (2.3), the misfit stress is short-range ( →1/*r*^3^).

#### (ii) Implementation into the three-dimensional DDD framework

To implement equation (2.3) into the DD framework, we need to transform the stresses from spherical coordinates to global Cartesian coordinates. According to second rank tensor transformation rules, the misfit stress field *σ*^*pt*^(*x*, *y*, *z*) at a point (*x*, *y*, *z*) in a Cartesian coordinate system can be written as

2.4 $$\begin{array}{r} {}[\sigma^{pt}]=\frac{2G\varepsilon R^{3}}{r^{5}}\frac{1+\nu }{3(1-\nu )}\left[\begin{array}{ccc} r^{2}-3x^{2} & -3xy & -3xz\\ -3xy & r^{2}-3y^{2} & -3yz\\ -3xz & -3yz & r^{2}-3z^{2} \end{array}\right]. \end{array}$$

Note that the above particle stress solution is for an isotropic particle in an isotropic matrix, which is consistent with the isotropic elasticity assumption used in the DD code. The stress field at any point in the simulation cell is the result of the superposition of the stress fields of the particles within the cell (plus contributions from the dislocation and the applied stress). Particle–particle interaction is not considered in the present work, which is an appropriate assumption for the dilute particle concentrations considered here, since the misfit stress decays quickly with distance from the particles.

The general situation analysed in the present work is schematically shown in figure 1. An arbitrarily shaped dislocation line is located near a spherical particle centred at *C*. Here, we only consider the situation of non-deformable and impenetrable particles; such that only the stress field outside of the particles needs to be accounted for. We will see in the following discussion that it proves convenient to isolate a dislocation segment, for example, segment *ij* bounded by nodes *i* and *j*, as demonstrated in figure 1. *X*_*i*_ and *X*_*j*_ are the coordinates of node *i* and *j*, respectively. *R* is the radius of the particle. To standardize the following computation, a local coordinate *s* is defined. The origin of *s* is located at the mid-point *O* of the segment, and the positive direction points from node *i* → *j*. *s* is normalized by half of the segment length *l*_*ij*_, *s* = 2*x*/*l*_*ij*_, such that *s* = −1 at the start node *i*, *s* = 1 at the end node *j*.

We define the coordinate of any point *s* along segment *ij* using shape functions (also known as interpolation functions) as follows:

$$x_{ij}(s)=[N_{i}\;N_{j}]\left[\begin{array}{c} X_{i}\\ X_{j} \end{array}\right],$$

where *s* ∈ [ − 1, 1], [*N*] = [*N*_*i*_ *N*_*j*_] is the one-dimensional linear shape function matrix

$$[N]=[N_{i}\;N_{j}]=\left[\begin{array}{cc} \frac{1-s}{2} & \frac{1+s}{2} \end{array}\right].$$

For dislocation lines located around the particles, the dislocation segment *ij* experiences a force per unit length that is proportional to the local misfit stress through the Peach–Koehler formula

2.5 $$f_{ij}^{pt}(x_{ij})=[\sigma^{pt}(x)\cdot b_{ij}]\times {\hat{l}}_{ij},$$

where **σ**^*pt*^(*x*) is the local stress at point *x* on the segment *ij*, ***b***_*ij*_ is the Burgers vector of segment *ij*, and ${\hat{l}}_{ij}$ is the local tangent vector of segment *ij*. Therefore, the contribution to the force on node *i* due to node *j* is

2.6 $$F_{i}^{ij}=\int_{0}^{l_{ij}}N_{i}f_{ij}^{pt}(x)\text{d}x=\frac{l_{ij}}{2}\int_{-1}^{1}N_{i}f_{ij}^{pt}(s)\text{d}s$$

with d*x* = (*l*_*ij*_/2) d*s*. In a simulation cell with multiple particles, the nodal force on node *i* caused by the misfit stresses is given by

2.7 $$F_{i}^{pt}=\sum_{I}\sum_{j}F_{i}^{ij},$$

where the inner summation is performed over all segments connected to node *i*, and the outer summation is over all particles in the simulation cell. To improve the computational efficiency, a 3-point Gaussian quadrature approach is used for the integration of equation (2.6), which was found to be precise and efficient when compared to evaluating the integral analytically. By substituting equation (2.7) into equation (2.1), we can construct the full nodal force ***F***_*i*_ that drives the dislocation motion.

### (b) Dislocation mobility law

Dislocation mobility is a fundamental property of crystals that determines most of the characteristics of their plastic deformation. It is usually modelled by a phenomenological law which prescribes the dependence of the steady-state velocity of a dislocation on local parameters, such as the stress, temperature, the line character (edge or screw) and the slip system.

#### (i) Dislocation glide

It is generally believed that, in FCC crystals, the interaction of phonons with dislocations provides the main mechanism of energy dissipation, so that dislocation glide can be considered an ‘automatically smooth’ process, particularly for a non-relativistic small dislocation velocity compared with the shear wave speed, and follows a linear phonon-drag law [37]. Whereas in BCC materials, the glide mobility is more complicated due to the non-planar character of the core of screw dislocations [40]. At stresses lower than the Peierls stress, which is defined as the minimum applied shear stress needed at 0 K to move a dislocation with infinite length over the periodic misfit energy barrier of the glide plane, dislocations move by the thermally assisted nucleation of kink-pairs and their subsequent migration. As the temperature and the applied stress increases, the Gibbs free energy for kink-pair nucleation vanishes, a dislocation moves as a whole again and experiences a friction force as a consequence of energy dissipation. In the present work, focus is limited to the dislocation behaviour at elevated temperature, where the phonon drag regime is predominant. A linear phonon-drag glide mobility law is therefore adopted here for BCC Fe,

2.8 $$V_{i}={\left(\frac{1}{2}\sum_{j}l_{ij}B_{ij}\right)}^{-1}F_{i},$$

where ***V***_*i*_ is the nodal velocity of node *i*, *l*_*ij*_ is the length of segment *ij*, ***B***_*ij*_ is the drag tensor determined according to the segment character, details of which are given in [4]. The sum is over all nodes *j* connected to node *i*, ***F***_*i*_ is the nodal mechanical driving force given in equation (2.1). Note that, in [4], following [37], a pure screw segment is assigned with an isotropic mobility in all directions perpendicular to the line, known as ‘pencil-glide’ behaviour. Such a treatment may exaggerate the cross-slip of screw dislocations, so that a more sophisticated cross-slip law is required.

#### (ii) Dislocation cross-slip

Cross-slip of screw dislocations is important in dynamic recovery of metals, which allows a dislocation to dissipate the maximum amount of plastic work from the system by allowing the dislocation to move on the glide plane that maximizes the area swept by its motion [41]. As with dislocation climb, the cross slip activity allows dislocations to move out of their original slip plane, which tends to make the substructure morphology appear cellular instead of planar. The cross slip process is more prolific in BCC materials owing to the availability of many secondary slip systems. It is therefore of significant importance to introduce a reasonable model to precisely describe the cross slip behaviour of screw dislocations. Pioneering studies have shown that the activation of cross slip depends critically on both the local stress state [42] and the dislocation line length [43]. Generally, the critical shear stress for cross-slip to occur increases with decreasing line length of the screw segment [43].

In the present work, dislocations gliding on {110}〈111〉 slip systems are considered, and cross slipping between these slip planes is allowed to occur when (i) the length of the screw segment exceeds a critical length *l*_*c*_, corresponding to the condition for stacking fault ribbon constriction [25]; (ii) the resolved shear stress on the cross-slip plane exceeds that on the current glide plane. We assume the same dislocation mobility on all the planes following [41,44].

#### (iii) Dislocation self-climb

At relatively lower temperatures dislocation cores can provide short circuit diffusion paths for atoms, which could accelerate the diffusion by more than three orders of magnitude compared to lattice diffusion. This allows dislocation motion perpendicular to the original slip system, known as dislocation self-climb, and is of particular importance in the low-temperature creep process and/or when the characteristic diffusion distance (such as the particle size in the current context) is small. Thus, in the present work emphasis is on the core-diffusion controlled self-climb motion in the single crystal.

In our recent work [4], a finite-element-based analysis of the dislocation core diffusion process is presented; based on a variational principle for the evolution of microstructure. The self climb model was then developed by implementing this core diffusion formulation into the nodal based three-dimensional DDD framework, the computational efficiency of which was further improved by implementing a paired-linear element based finite element discretization method [35]. In the improved self-climb model [35], dislocations are discretized into a series of straight segments (one-dimensional linear elements). The climb velocity is defined at each node and varies linearly along the segment, and the diffusive flux is also defined at each node. A series of Lagrange multiplier are introduced to enforce the flux continuity at triple or quadruple junction nodes (dislocation nodes connected with more than two arms). A set of linear simultaneous equations is then derived as the kinetic equations for self-climb,

2.9 $$\left[\begin{array}{cc} \left[K\right] & {\left[C^{s}\right]}^{T}\\ \left[C^{s}\right] & \left[0\right] \end{array}\right]\left[\begin{array}{c} \left[V_{c}\right]\\ \left[\lambda \right] \end{array}\right]=\left[\begin{array}{c} \left[F_{c}\right]\\ \left[0\right] \end{array}\right],$$

where [*K*] is the global viscosity matrix for core diffusion, [*C*^*s*^] is a constraint matrix, [0] is a *N* × *N* null matrix, where *N* is the number of junction nodes. [*V*_*c*_] is the vector of unknowns, including the nodal climb velocities and fluxes, [*λ*] is the vector of Lagrange multipliers, [*F*_*c*_] is the vector of generalized nodal forces. The nodal climb velocity can then be derived by solving equation (2.9). To bridge the large time scale separation between glide and climb motion, as stated in the Introduction, an adaptive time scheme [4] is adopted here. In which, dual time increments are adopted for the glide and climb steps as follows. We apply the stress and initially perform the simulation using a small time increment of ∼1 ns to resolve glide/cross-slip-related events, until the plastic strain rate caused by dislocation glide/cross-slip approaches zero. We then stop the glide/cross-slip motion, and compute the evolution by diffusion by solving equation (2.9) to derive the climb velocity. At this point, a much larger value of time increment, depending on the effective core diffusivity and temperature, is used. Once the dislocation climbs to a neighbouring slip plane and allows further glide/cross-slip, the climb process is stopped and the glide/cross-slip motion is activated again. For more details about the self-climb model and the adaptive time scheme, refer to [4,35].

### (c) Finite boundary conditions

Simulating micromechanical tests requires modelling a finite domain with complex geometry and boundary conditions, which can be generally achieved by coupling with a finite-element method (FEM). The coupling procedures are mainly divided into two categories: the discrete-continuous method [45], in which the plastic strain generated by dislocation motion is used in the elastic FE code to solve the total stress; and the superposition method [46], which uses the superposition principle of [47] to enforce the desired traction and displacement boundary values. In the present work, the superposition method is adopted. In this method, a finite-element mesh is constructed firstly, corresponding to the desired finite domain. Field quantities in this finite domain are partitioned as

2.10 $$\sigma =\tilde{\sigma }+\hat{\sigma },\varepsilon =\tilde{\varepsilon }+\hat{\varepsilon },u=\tilde{u}+\hat{u}$$

where **σ**, **ε** and ***u*** are the total stress field, strain field and displacement field, respectively. The tilde ( ~ ) denotes the infinite-body fields calculated from the superposition of the infinite analytical fields of all dislocations in the domain, and the hat ( ^ ) fields are smooth complementary fields that enforce the boundary conditions for a specified problem. As in any mechanical finite-element problem, with specified traction ***T*** or ***U*** applied on the surface, every FE surface node is in parts of the boundary over which tractions ***T*** or displacements ***U*** are applied. At each time increment, the tractions, $\tilde{T}$, and the displacements $\tilde{U}$, arising from the dislocation network in an infinite medium are evaluated analytically at the surface FE nodes, respectively. Modified tractions and displacement boundary conditions $\hat{T}=T-\tilde{T}$, $\hat{U}=U-\tilde{U}$ are then used in an elastic FE code to derive the corrective fields $\hat{\sigma }$, $\hat{u}$ and $\hat{\varepsilon }$. The corrective stress field, $\hat{\sigma }$, is then used to calculate the dislocation nodal force shown in equation (2.1) in a finite volume. It is worth mentioning that, in the present work, a newly developed method [48] for accurately calculating the displacement field produced by an arbitrary dislocation network on the surface of a finite domain, is adopted, which has been proved to be readily compatible with DDD codes and allows for easy parallelization [29].

Before closing this section, we would like to comment on a subtle issue about the calculation of displacement field in an infinite volume $\left(\tilde{u}\right)$ when considering dislocation glide, climb and cross-slip altogether. There is a concern about this issue in the literature that the simple superposition of analytical displacement fields of dislocations cannot properly treat the displacement discontinuities caused by the out-of-plane climb motion [49,50]. Thus, it is believed that in a combined glide and climb process, information about the dislocation slip area history has to be recorded and stored at each time increment to calculate the displacement field accurately. However, in the method proposed in [48], an adapted Barnett solution [26] is developed, to calculate the displacement contribution of a single finite straight dislocation segment, which can be easily implemented into a three-dimensional dislocation dynamics code without the need to store any history information. Thus, the average surface displacement $\tilde{U}$ in a uniaxial specimen caused by the overall internal dislocation motion can be calculated directly from the total area swept by the dislocations, and it does not matter where in the volume that slip or climb occurs. This may seem counterintuitive because the plasticity caused by dislocation motion should be path-dependent. The paradox does not really exist here, as we only need to find the average displacement on the surface.

To clarify this point, a relatively straightforward analysis of the surface displacement caused by internal plastic deformation as a result of dislocation motion is given in appendix A. From which, we can see that, the surface displacement is determined only by the amount of slipped area (swept out by the dislocations) and not to the current position of the dislocations—we only need to know the amount of glide and climb, not the route followed to get there.

## 3. Dislocation–particle interactions

The addition of a particle dispersion to metals is a common strategy adopted to strengthen alloys. When a gliding dislocation approaches an obstacle intersecting its glide plane, it becomes pinned and bows around the obstacle. At a critical unpinning stress, the dislocations are able to loop around the obstacles and pinch off, continuing their motion whilst leaving behind dislocation shear loops encircling the particles, as demonstrated originally by Orowan [10]. At applied stresses below the Orowan stress, pinned dislocation can still unpin from the obstacles by thermally activated mechanisms, such as dislocation climb and cross-slip. These unpinning processes represent typical microstructural features mediating plastic deformation, particularly during loading at elevated temperature. In this section, we simulate two typical particle bypass mechanisms based on the new method developed in §2. The examples described in the following subsections are chosen to illustrate the proposed method, and to demonstrate that it produces accurate results by comparison with analytical results [12], available numerical simulations [22,41,51] and experimental observations [52]. Parameters for bcc Fe used in the following simulations are shown in table 1.

**Table 1.** Parameters for *α*-iron.

parameters	magnitude
shear modulus	*G* = 83 GPa
Poisson’s ratio	*ν* = 0.29
lattice parameter	*a* = 0.2856 nm
Burgers vector	$b=\sqrt{(}3)a/2$
dislocation core radius	*r*_*c*_ = 5*b*
pre-exponential for core diffusion	$a_{\text{core}}D_{\text{core}}^{0}=1\times 10^{-23}\;{\text{m}}^{4}\;{\text{s}}^{-1}$ [53]

### (a) Punching out prismatic dislocation loops

Elastic accommodation and punching of prismatic dislocation loops (PDLs) have been experimentally observed around sub-micrometre-sized particles [52], which are assumed to form through a dislocation cross-slip mechanism. The classical model for PDL generation around misfit particles was proposed by Ashby & Johnson [12], and later confirmed by means of nonlinear elastic/phase-field simulations [51], atomistic simulations [54] and dislocation dynamics simulations [55]. As illustrated in Ashby & Johnson’s model, when the misfit in the particle–matrix interface exceeds a critical value, a dislocation segment bulges from the interface on a slip plane where the shear stress is greatest. The maximum shear stress has been proved to be created at the particle–matrix interface on the {111} plane intersecting the spherical particle at a height of $R/\sqrt{2}$ [54] in FCC aluminium. Here we explore the availability of cross-slip planes on PDL generation in a BCC lattice. The family of bcc slip systems considered is {110}〈111〉.

**Figure 2.**
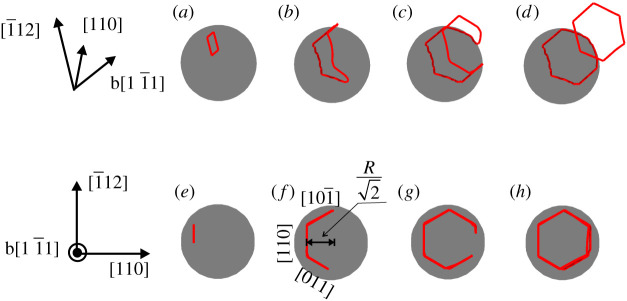
Snapshots during the punching out of an interstitial-type prismatic dislocation loop (PDL) near a spherical particle in a bcc lattice. Two views are given at each stage: the upper sequence, (*a*–*d*), provides a profile of the PDL as it peels off from the particle’s surface; the lower sequence, (*e*–*h*), is viewed normal to the dislocation’s Burgers vector, $b=[1\bar{1}1]/2$, providing a view of the whole loop. (Online version in colour.)

In the present work, attention is confined to the formation of a PDL from a shear loop by cross-slip; nucleation of the shear loop at the interface is therefore not considered and the simulation begins with a given incipient dislocation shear loop. In figure 2*a* and *e*, we show the initial dislocation shear loop placed on the BCC $\left(110)[1\bar{1}1\right]$ slip system from two views, and the habit plane of the loop is placed at a height of $R/\sqrt{2}$ near the particle. Radius of the particle is *R* = 20 nm and the side of the initial square shear loop is set as *L* = 10 nm. Other parameters needed are shown in table 1. In the absence of an applied stress, this incipient shear loop is then allowed to evolve under the misfit stress field. Snapshots of major stages during the evolution are demonstrated in figure 2. Following [41], two views are provided at each stage. The upper sequence, figure 2(*a*)–(*d*), gives the view of two PDLs formed at the particle; while the lower sequence, (e)–(f), provides the projection normal to the Burgers vector $b=[1\bar{1}1]/2$. A movie is available in electronic supplementary material, Movie S1.

As illustrated in figure 2, the shear loop expands to increase the length of screw segments, creating a highly probable situation for the dislocation to cross slip to another highly stressed slip plane. The screw dislocations are then driven around the particle by multiple cross-slip, to meet dislocations of opposite line directions, resulting in pinch-off on the (110) slip plane, so that two PDLs are formed: one vacancy-type prismatic loop surrounding the particle and one interstitial-type loop punched out from the particle; the former is attracted to the particle, reducing the misfit strain, while the latter is repelled. This series of events is consistent with the theoretical prediction from [12] and the numerical simulation results from [41,51].

In fact, the competition between cross-slip of a screw segment and glide of the edge segment determines whether a PDL or a helical dislocation would form: a PDL forms when the cross-slipped segments meet their partners of opposite sign and pinch off, which is promoted by the symmetry in the misfit stress field and the initial dislocation configuration; a helical dislocation is formed if the cross-slipped screw segments miss each other when the symmetry is broken, which give rise to a long spiral to produce a continuous emission of PDLs, as observed in indentation [56] and precipitation [52] experiments.

### (b) Particle bypass by self-climb mechanism

**Figure 3.**
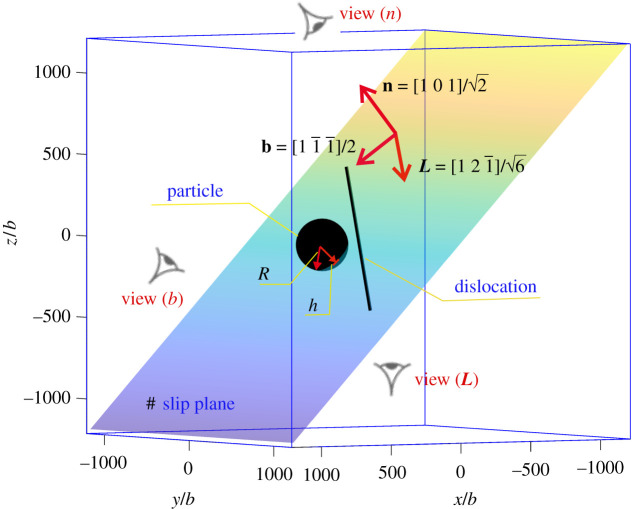
A dislocation interacts with a particle by the glide plus climb mechanisms. **n**, **L** and **b** are the plane normal, dislocation line vector and Burgers vector, which are the line-of-sight vectors for the camera views of View (**n**), View (**L**) and View (**b**), respectively. (Online version in colour.)

Dislocation self-climb plays a significant role in dislocation creep behaviour of particle strengthening materials. In this subsection, we examine the dislocation self-climb mechanism by which dislocations surmount particles. We simulate the motion of an initially straight pure edge Frank–Read source (FRs), i.e. a straight edge dislocation pinned at two ends, moving towards a spherical impenetrable particle under an applied stress lower than the Orowan shear stress *τ*_*c*_, so that dislocations cannot bypass the particle without climb or cross-slip. A general situation considered here is depicted in figure 3. The size of the simulation box is set as 600 nm × 600 nm × 600 nm, to make sure the surfaces are far from the internal configuration, so that surface effects are negligible. The length of the initial FRs is *L* = 490 nm with a line vector along $L=[12\bar{1}]/\sqrt{6}$. The normal of the slip plane where the FRs lies is $\text{n}=[101]/\sqrt{2}$, and the Burgers vector is $\text{b}=[1\bar{1}\bar{1}]/2$. The particle intersects the slip plane with a radius *R* = 40 nm. The applied shear stress is set to be 0.6*τ*_*c*_. Dislocations will then evolve as a result of the competition between the applied stress, the line tension and the misfit stress. Two key variables that determine the bypass mechanism are considered here, the dilatational misfit strain ε and the distance from the centre of the particle to the slip plane containing the dislocation line *h*. Three typical cases are discussed here: in case I, the dilatational misfit strain ε is set to zero, ε = 0, and the initial FRs intersects the particle at $h=3R/4\sqrt{2}$; in case II, we increase the misfit stress by setting ε = 0.001, while retaining the relative position between the particle and the dislocation, i.e. $h=3R/4\sqrt{2}$; in case III, we keep the misfit strain at ε = 0.001, and decrease the distance between the particle centre and the intersecting plan to $h=R/2\sqrt{2}$, i.e. the dislocation lies on a slip plane closer to the equator of the particle. For all cases, the applied stress remains the same. Temperature *T* = 800 K, at which the diffusion process is dominated by core diffusion rather than lattice diffusion [57].

#### (i) No misfit stress

**Figure 4.**
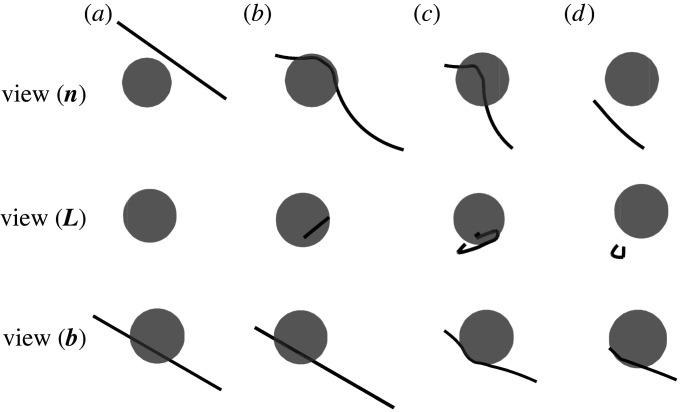
Snapshots during case I: ε = 0, $h=3R/4\sqrt{2}$. The dislocation climbs under the bottom of the particle by a glide plus self-climb mechanism. (Online version in colour.)

In case I, there is no misfit strain, ε = 0, so that the dislocation moves towards the impenetrable particle under the applied shear stress. Simulation results are presented in figure 4, in which typical snapshots during the evolution are demonstrated from three lines of sight. View (***n***), view (***L***) and view(***b***) are the lines of sight according to the plane normal ***n***, initial dislocation line vector ***L*** and Burgers vector ***b***, respectively, as illustrated in figure 3. The particle in figure 4 is set as translucent to show the dislocation structures beyond it. Initially, the edge dislocation is straight, lying in the slip plane below the equator of the particle at $h=3R/4\sqrt{2}$. The dislocation approaches the particle and then bends around it by gliding on its original slip plane. Rather than Orowan looping, the curved dislocation is then trapped by the particle, as shown in figure 4*b*, because the applied stress is not high enough to drive the dislocation further to complete the Orowan process. Following the adaptive time scheme [4] adopted in the current simulation, the climb motion is activated once the glide process stops. The curved dislocation segments around the particle then move by ‘local climb’, normal to the original slip plane, to an adjacent slip plane and leave the particle surface. This climb motion gives rise to fluctuations of the dislocation line in order to conserve mass. Compared to a pure edge segment, a mixed dislocation segment climbs a larger distance when consuming the same volume of material. As a result, as the mixed dislocation segments which profile the particle climb downwards, the near-edge segments at the sides climb upwards by a smaller amount. This allows the climbed segments to glide again to profile the particle surface. This process then repeats, until the dislocation climbs under the particle and does not leave a dislocation loop behind, as shown in figure 4*c*–*e*. A movie is available in electronic supplementary material, movie S2. Note that, in this process, the core diffusion distance is relatively short, with atoms only transported local to the particle, so that other segments far from the particle remain in their original slip plane. This particle bypass mechanism is believed to play a significant role in plastic recovery by facilitating the annihilation of Orowan loops at the particle interface [5]. A similar climb process is observed in [22]. Note that the cross-slip process is excluded since the dislocation is mainly edge/mixed character, so that for this situation the glide plus self-climb mechanism dominates during the bypass process.

#### (ii) With misfit stress

**Figure 5.**
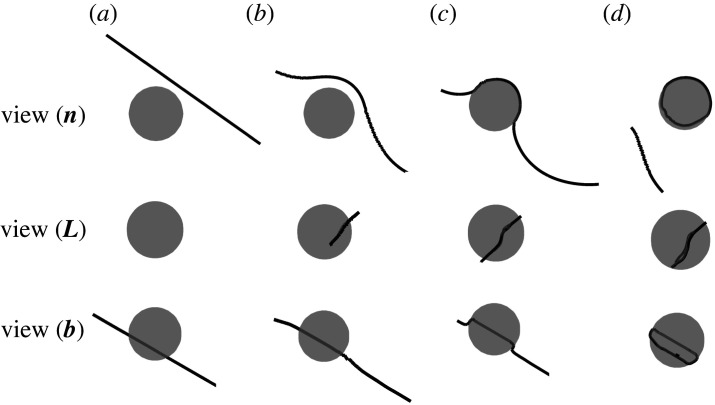
Snapshots during case II: ε = 0.001, $h=3R/4\sqrt{2}$. The dislocation bypasses the particle and a shear loop is left around the particle.

In case II, we consider particle bypass when there is a misfit strain between the particle and matrix. The misfit strain is ε = 0.001, other parameters remain the same as in case I. Simulation results are presented in figure 5. We can see that, initially, the dislocation glides towards the particle under the applied stress. As the dislocation approaches the particle, the misfit stress increases rapidly to become dominant and eventually repels the dislocation, so that the glide velocity decreases and the dislocation stands off from the particle as it begins to bend around it, as shown in figure 5*b*. Climb is activated once the applied stress is balanced by the misfit stress and the glide motion stops. The dislocation segments around the particle then climb, driven by the misfit stress perpendicular to the slip plane. As demonstrated in figure 5*c*,*d*, the edge segments climb upwards while approaching the particle surface, whereas the mixed segments climb downwards. Note that, although the misfit stress repels the dislocation as it moves towards the particle, it attracts the segments once the dislocation bows out on the other side of the particle, owing to the symmetry of the misfit stress as stated in §2(i) (resulting in the Peach–Koehler force on the segments caused by the misfit stress changing sign). We can see from the movie provided in electronic supplementary material, movie S3, once the dislocation bows out to the other half of the particle, it loops around the surface of the particle very quickly due to the attraction from the misfit stress. When the total stress acting on the dislocation line is high enough to balance the line tension caused by the bow-out, the dislocation keeps bowing and pinches off to bypass the particle, leaving an inclined shear loop. This shear loop will annihilate by the self-climb plus glide process as illustrated in case I when repelled by an incoming dislocation.

#### (iii) With/without cross-slip

**Figure 6.**
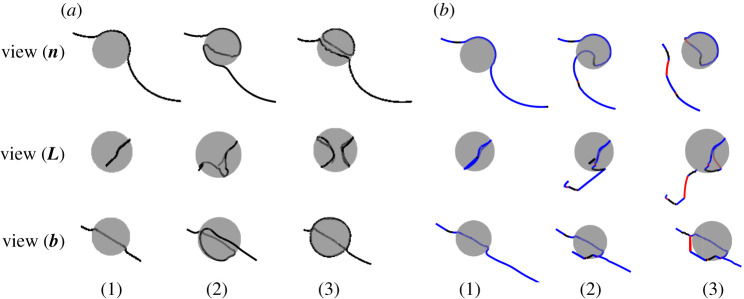
Snapshots during case III: ε = 0.001, $h=R/2\sqrt{2}$. (*a*) when the cross-slip mechanism is disabled, dislocation is trapped by the particle; (*b*) when cross-slip is enabled, dislocation bypass the particle and a semi-prismatic loop is left around the particle. (Online version in colour.)

We now turn to case III, when the initial FRs lies in a slip plane closer to the equator of the particle with $h=R/2\sqrt{(}2)$, so that a higher stress is needed to drive the dislocation to bow-out and bypass the particle. Other parameters remain the same as in case II. At first, the edge dislocation moves towards the particle by gliding on its slip plane and is repelled by the misfit stress as it approaches the particle surface; climb is then activated when the glide motion is stopped. This evolution process is similar to case II. However, unlike case II, as the dislocation moves around the particle to the other side, the total stress (composed of the applied stress, the misfit stress and the line tension) acting on the dislocation is not high enough to facilitate bowing out to form a shear loop. Dislocations will then be trapped by the particle. Two situations are considered here, and the corresponding results are demonstrated in figure 6*a*,*b*. When cross-slip of screw dislocation segments is disabled, edge segments around the particle will climb until they are trapped at the equator of the particle, where the component of the nodal force in the climb direction is zero. As shown in figure 6*a* 1–3, the mixed dislocation keeps moving downwards by the glide plus climb process until it pinches off at the bottom of the particle, while adjacent segments move in the opposite direction to conserve mass. This leads to the formation of a dislocation loop composed of a semi-circular prismatic loop and semi-shear loop around the particle. The other half of the prismatic loop is attracted to the surface of the particle and pinned at the leaving side due to the misfit stress, which is consistent with the theoretical analysis in [19]. This dislocation–particle interaction is essentially different from the particle bypass processes shown in cases I and II. A movie is provided in electronic supplementary material, movie S4. While, if cross-slip is enabled, as the dislocation bows out and increases the length of the screw segments, the misfit stress drives the screw segments of the bowed dislocation to cross-slip on the sides of the particle, as illustrated in figure 6*b* 1–3. The cross-slipped dislocation segments then pinch-off near the surface of the particle and glide away from the particle, leaving a semi-hexagonal prismatic loop around the particle. A movie is provided in electronic supplementary material, movie S5. Similarly, if a higher shear stress is applied, the dislocation could bypass the particle more quickly by a glide plus cross-slip mechanism, as shown in electronic supplementary material, movie S6.

The bypass processes presented above indicates that the particle bypass mechanism is sensitive to the position where the dislocation intersects the particle, and the evolution process is strongly influenced by the misfit stress. The symmetry and strength of the misfit stress are responsible for many of the new mechanisms observed during the interaction between the dislocation and the particle, which are significantly different from those seen in the classical bypass processes for non-misfitting particles, leading to either a decrease or an increase in the minimum stress required to bypass the particles. Dislocation self-climb and cross-slip mechanisms are critical in the particle bypass process when the applied stress is much lower than the Orowan shear stress. These are essentially independent processes, but the above examples demonstrate how they can combine to allow dislocation to bypass particles. At low stresses self-climb dominates. A feature of this mechanism is that no dislocation structures are left surrounding the particle after the bypass process. At high stress, cross-slip dominates and prismatic loops are generated around the particle as it is bypassed. At intermediate stresses, we observe a combination of these processes—for example, self climb of a dislocation segment can lead to the formation of screw segments that can subsequently cross-slip, or the climb process can promote glide of another part of the dislocation to glide into a new configuration that is favourable for cross-slip. We see similar type of processes during creep of a body that contains multiple particles and dislocations. This situation is examined in the following section.

## 4. Creep test during compression of micropillars

In the previous sections, we studied the simple cases of the interaction between a dislocation and an individual particle by the combination of glide, cross-slip and self-climb mechanisms. For these cases, we can readily observe how the misfit stress, the coupled glide/cross-slip and glide/self-climb mechanisms influence the dislocation evolution around an individual particle, which further validates the model developed in §2. We now move on to simulate the collective dislocation behaviour in the presence of a cluster of particles, to investigate the emergent interactions and larger-scale-patterning in BCC iron, based on the local rules discussed above.

### (a) Simulation set-ups

**Figure 7.**
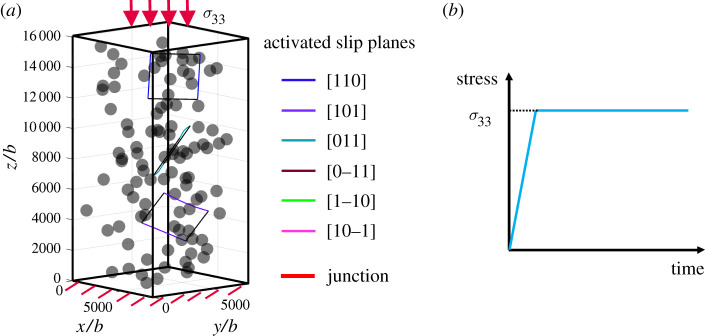
(*a*) Initial simulation set-ups of the compression test. Dislocation segments on different slip panes are assigned with different colours, and junctions are highlighted with bold line. (*b*) Schematic illustration of the loading history during compressive loading. (Online version in colour.)

The simulations are performed within a *α*-Fe single crystal micropillar, as shown in figure 7. The size of the simulation box is 2 *μ*m × 2 *μ*m × 4 *μ*m, with the bottom surface fixed and compression applied on the top surface along the [001] direction. The four sides are free. Dislocations can exit freely from all the surfaces except the bottom. Impenetrable and non-deformable particles are randomly distributed inside of the box. The volume fraction of particles is set as 0.02. Particles are mono-sized spheres with radius *R* = 80 nm. The misfit strain is set to be ε = 0.001. Three prismatic loops are introduced as the initial dislocation configuration, which are dispersed randomly inside of the simulation cell and assigned with different slip planes. Two sides of each loop are pinned, as indicated by the black segments shown in figure 7*a*, so that each prismatic loop provides a pair of FRs. The initial FRs are located on [110] (blue segments), [101] (purple segments) and [011] (bluegreen segments) slip planes, respectively. The length of the FRs are set as *L* = 400 ± 50 nm. Dislocations are allowed to glide or cross-slip on all planes of the {110}〈111〉 slip system at temperature *T* = 900 K. Here, *α*-Fe is approximated to be elastically isotropic, and general material properties are shown in table 1.

Dislocation behaviour during the creep test under different compression stresses are simulated here. In each simulation, the applied stress is slowly ramped up from 0 to the specified stress level *σ*_33_, and then held constant, as schematically illustrated in figure 7*b*, to investigate dislocation evolution during the constant-stress creep process. Results under different stresses are presented and discussed in the following subsection.

### (b) Results and discussion

**Figure 8.**
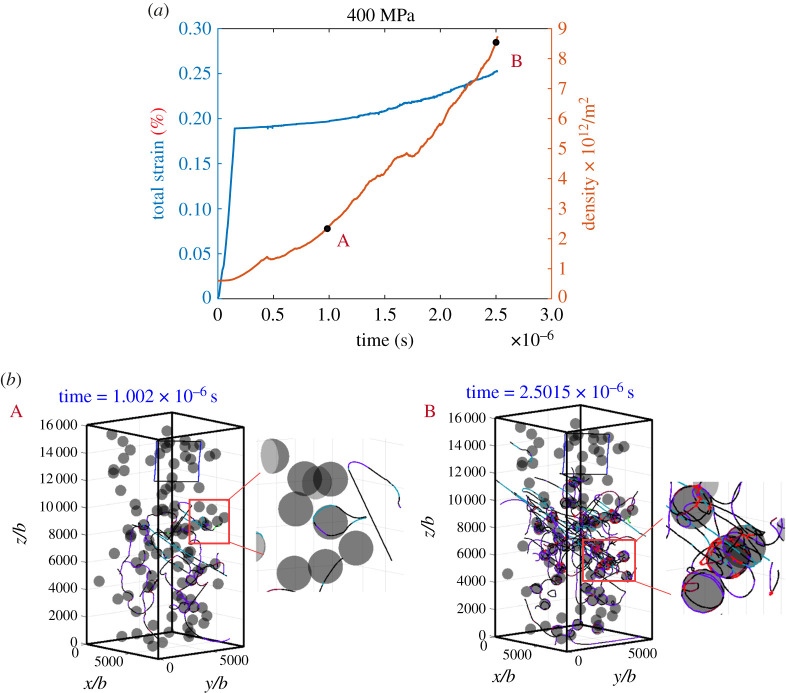
Simulation results under a compressive stress of 400 MPa: (*a*) total strain–time curve and dislocation density–time curve; (*b*) snapshots of typical dislocation configurations during evolution corresponding to the marked dots in (*a*), in which structures in the snapshots as labelled with a box are further magnified. (Online version in colour.)

The simulation result for creep of an *α*-iron micropillar under a constant stress of *σ*_33_ = 400 MPa, is demonstrated in figure 8. Evolution of the total strain and dislocation density over real time are plotted in figure 8*a*. Note that the unit of the strain is %. Snapshots of typical dislocation configurations during the evolution corresponding to the marked points A and B are shown in figure 8*b* and representative dislocation structures from the red boxes are shown magnified adjacent to the micropillar. A movie showing the evolution is provided in electronic supplementary material, movie S7. From the strain–time and dislocation density–time curves, we can see that, the strain rate increases rapidly as the mobile dislocation density increases, which occurs over a time scale of approximately microsecond, indicating that the dislocation multiplication is driven by glide or cross-slip mechanisms. This is illustrated more straightforwardly by the dislocation structures corresponding to the marked points A and B, as shown in figure 8*b*. Dislocation structures at point A are wavy-like, and the dislocation loop around the particle in the magnified region is sited on different slip planes, indicating obvious cross-slip events. As the dislocation density increases to point B, many dislocation loops form around the particles, lying on different slip planes, as a result of combined glide and cross-slip mechanisms as a series of gliding dislocations bypass the particles. The interaction between the loops and mobile dislocations leads to the formation of junctions on the surface of the particles, as illustrated in the red box in figure 8*b*(B) (junctions are highlighted in red). The resultant dense interfacial dislocation network enveloping the particles is believed to resist further plastic flow and contributes to the hardening of particle-strengthened alloys [58].

**Figure 9.**
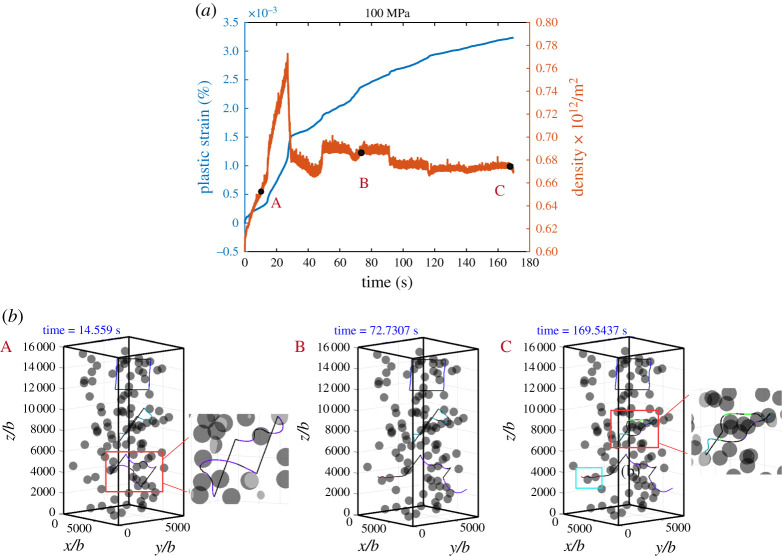
Simulation results under a compressive stress of 100 MPa: (*a*) strain–time curve and dislocation density–time curve; (*b*) snapshots of typical dislocation configurations during the evolution corresponding to the marked dots in (*a*), and the dislocation structures in the boxes in the snapshots are further magnified. (Online version in colour.)

In case II, we consider the creep process with a much lower applied stress, *σ*_33_ = 100 MPa. The plastic strain–time curve and dislocation density–time curve are plotted in figure 9*a*, and the dislocation structures with respect to the marked points A, B and C on the curves are demonstrated in figure 9*b*. A movie is provided in electronic supplementary material, movie S8. The most remarkable difference is that the deformation occurs over a much longer time-scale, approximately 200 s, resulting in a strain rate much lower than that at 400 MPa, indicating that the diffusion-controlled climb mechanism plays a significant role in this process. At first, dislocations from the initial FRs glide and bow out under the applied stress, leading to a sharp increase in the plastic strain and the dislocation density. The bowed dislocations are then blocked by the particles as shown in figure 9*b*(A). Since the dislocation characters are mainly edge or mixed type, the blocked dislocations then move by a glide plus self-climb process to bypass the particles, following the path shown in figure 4. This leads to a decrease in the strain rate since climb occurs over a much longer time scale than glide. Once a blocked dislocation is released from a particle, glide becomes dominant and the strain rate increases, until it is blocked again by other particles, as demonstrated in 9(*b*).B. The sharp decrease in the dislocation density is caused by the dislocations exiting from the surface of the pillar. Also, the climb dominated bypass mechanism does not leave any dislocation around the particles to contribute to the dislocation and obstruct the motion of subsequent dislocation gliding through the material. As shown in figure 9(*b*)(C), dislocations trapped by a particle try to bypass by self-climb and shrink, leading to a gradual decrease in the dislocation density. We can see that, at a very low stress level, although the plastic deformation is generated mainly by glide and cross-slip, the overall strain rate is controlled by the self-climb process required to overcome the particles. When the out-of-plane motion is activated, dislocations can evolve to a lower energy state by local climb, even when a very low stress is applied.

**Figure 10.**
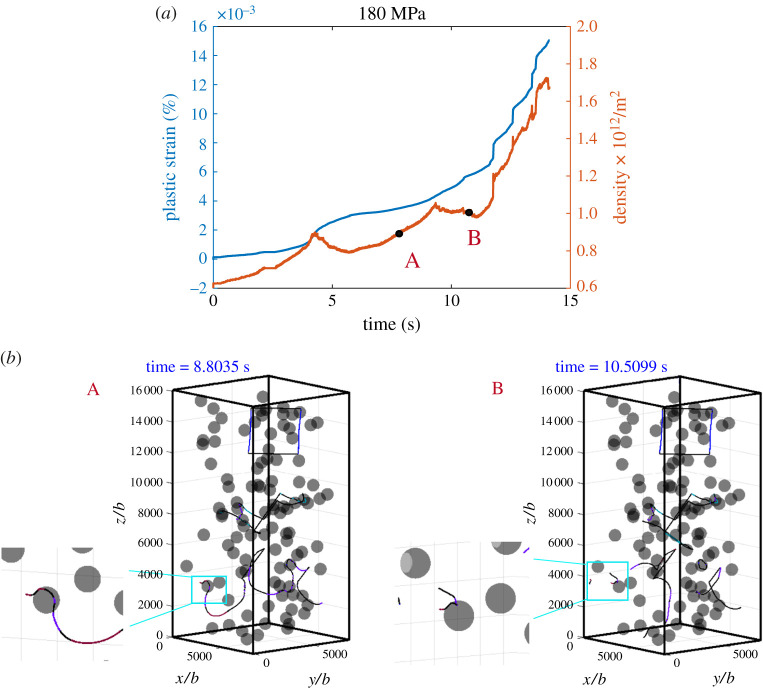
Simulation results under a compressive stress of 180 MPa: (*a*) plastic strain–time curve and dislocation density–time curve; (*b*) snapshots showing typical dislocation configurations during the evolution corresponding to the marked dots in (*a*). (Online version in colour.)

**Figure 11.**
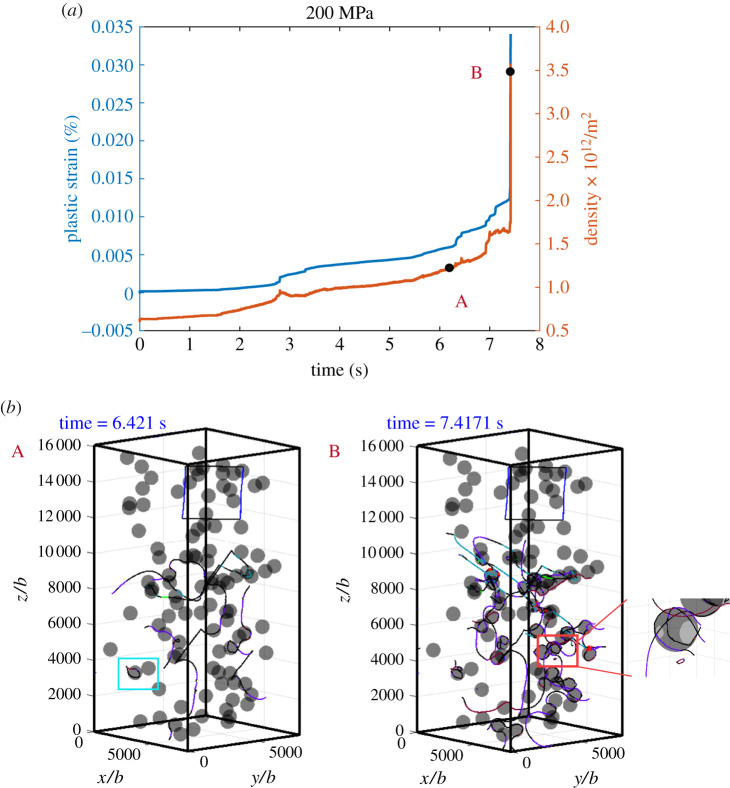
Simulation results under a compressive stress of 200 MPa: (*a*) strain–time curve and dislocation density–time curve; (*b*) snapshots of typical dislocation configurations during the evolution corresponding to the marked dots in (*a*). (Online version in colour.)

We now turn to the intermediate stress level, *σ*_33_ = 180 MPa. Simulation results are shown in figure 10. A movie is provided in electronic supplementary material, movie S9. The plastic strain rate exhibits a gradual increase as the dislocations overcome the particles and bow out to increase the dislocation density. These events span over a time scale of approximately 10 s, which sits in between case I and case II, demonstrating that deformation occurs by a combination of glide, cross-slip and climb. This is illustrated by the dislocation configurations corresponding to the marked points A and B as shown in figure 10*b*(A,B), where the most noticeable feature is labelled in the zoomed in cyan box. Note that, at low applied stress in case II, dislocations are pinned by the particles as labelled in the cyan box in figure 9(*b*)(C). As the applied stress increases to 180 MPa, the pinned dislocations can bow out, as a dislocation climbs to the top of the particle, screw segments form and the particle is bypassed by cross-slip, leading to a sharp increase in the dislocation density and plastic strain rate. As we further increase the applied stress to 200 MPa, a pinned dislocation can easily bypass the particle by Orowan looping, as illustrated in the cyan box labelled in figure 11(*b*)(A), giving rise to a higher rate, due to the shorter waiting time at bypassing particles. The dislocation density also increases more quickly. The other important feature in the dislocation configuration is shown in figure 11(*b*)(B), a prismatic loop is formed and is punched out from the surface of a particle. Multiple dislocation shear loops are left around the particle distributed parallel to each other. This is consistent with the evolution processes prescribed in §3a and b(i), suggesting that, at intermediate stress levels, dislocation evolution is dominated by a combination of glide, cross-slip and self-climb. As more and more dislocations overcome the particles, a relatively dense dislocation network develops and the mobile dislocation density increases, so that there is enough dislocation motion to maintain a sustainable plastic deformation; glide/cross-slip becomes dominant again and the strain rate increases rapidly. A movie is provided in electronic supplementary material, movie S10.

## 5. Conclusion

Creep deformation has long been a concern for engineers—particularly in the power generating industries. As summarized in the deformation mechanism maps by Frost & Ashby [53], creep of a real alloy is a rather complex phenomenon controlled by several competing deformation mechanisms such as dislocation glide, climb, cross-slip, multiplication through Frank–Read and other types of sources, and short-range interactions including dislocation annihilation, jog and junction formation, point defect diffusion and dislocation–particle interactions, bringing great challenges to the experimental and numerical investigations.

To our knowledge, we present here the first three-dimensional DDD framework for particle strengthened materials that account for dislocation glide, cross-slip and self-climb mechanisms. Coupled with a superposition method, it provides an effective model to predict the creep behaviour of particle-strengthened materials within a finite domain at relatively high temperature, by revealing the collective dislocation behaviour in the presence of particles.

The dislocation interaction with individual particles is systematically studied first. Our simulation results suggest that self-climb plays a decisive role in the particle bypass processes when the applied shear stress is much lower than the Orowan shear stress. The dislocation evolution process is strongly influenced by the misfit stress, the symmetry and strength of which is responsible for many of the new mechanisms observed during the dislocation–particle interactions, leading to either a decrease or an increase in the minimum stress required to bypass the particles.

The collective dislocation behaviour in the presence of a cluster of particles are further simulated to investigate the constant-stress creep deformation of particle-strengthened bcc micropillars. As mentioned above, creep due to dislocation motion involves a number of processes, many of which must occur sequentially. As a result, the slowest of these processes usually controls the overall rate of deformation. Our simulations reveal three typical categories of plastic deformation: at a relatively high stress level, dislocation evolution is dominated by dislocation multiplication driven by glide and cross-slip, giving rise to a dense interfacial dislocation network enveloping the particles. At a much lower stress level, dislocations are held up by the particles and dislocation climb plays a decisive role in bypassing the particles, so that the overall strain rate is controlled by the climb process, although the plastic deformation is mainly generated by the glide/cross-slip processes. The strain rate is, therefore, much lower than that at high stress levels. At an intermediate stress level, it is the combination of glide, cross-slip and self-climb mechanisms that dominates the dislocation evolution. Furthermore, in line with the dislocation interactions with individual particles described in §3, multiple particles in the micropillar provide more complicated stress fields for the surrounding dislocations, and give rise to unique dislocation behaviour as a result of the local evolving driving forces in combination with the different types of dislocation motion.

Our investigation in the present work enables a systematic interpretation of the particle bypass mechanisms and provides an in-depth mechanistic understanding of the collective dislocation behaviour at relatively high temperature, extending our knowledge of creep deformation of particle-strengthened alloys.

## Figures and Tables

**Figure 12.**
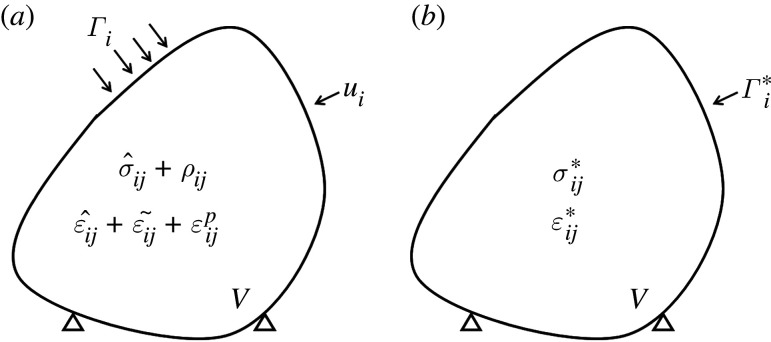
A schematic illustration of the considered volume.

## Data accessibility

The code used for the discrete dislocation dynamic simulations is freely available from https://github.com/TarletonGroup/EasyDD. Additional supporting data are available as electronic supplementary materials.

## Appendix A. Surface displacement as a result of internal plastic deformation

Consider a body of volume *V*, which experiences plastic strain internally, $\varepsilon_{ij}^{p}$, under load, i.e. surface traction $\Gamma_{i}$, as schematically shown in figure 12*a*, the stress field is

1 $$\sigma_{ij}={\hat{\sigma }}_{ij}+\rho_{ij},$$

where ${\hat{\sigma }}_{ij}$ is the stress field in equilibrium with $\Gamma_{i}$ given by an elastic analysis and *ρ*_*ij*_ is a residual stress field that gives rise to additional elastic strain ${\tilde{\varepsilon }}_{ij}$, such that

2 $${\tilde{\varepsilon }}_{ij}=C_{ijkl}^{-1}\rho_{kl},$$

where $C_{ijkl}^{-1}$ is the elastic compliance matrix. Note the elastic strain field arising from ${\hat{\sigma }}_{ij}$ is

3 $${\hat{\varepsilon }}_{ij}=C_{ijkl}^{-1}{\hat{\sigma }}_{kl},$$

where ${\hat{\varepsilon }}_{ij}$ is a compatible strain field, but ${\tilde{\varepsilon }}_{ij}$ is generally not. This field is generated to accommodate the plastic strain $\varepsilon_{ij}^{p}$, such that

4 $$\varepsilon_{ij}={\hat{\varepsilon }}_{ij}+\varepsilon_{ij}^{p}$$

is compatible.

Here we consider the situation where the body is elastically homogenious, i.e. *C*_*ijkl*_ is the same everywhere.

Now consider a point on the surface of the body. We wish to determine the displacement at this point. The displacement *u*_*i*_ has two components

5 $$u_{i}={\hat{u}}_{i}+u_{i}^{p},$$

where ${\hat{u}}_{i}$ is the elastic displacement due to $\Gamma_{i}$ and $u_{i}^{p}$ is the displacement arising from the plastic strain $\varepsilon_{ij}^{p}$ and the residual strain ${\tilde{\varepsilon }}_{ij}$.

Note ${\hat{u}}_{i}$ is compatible with ${\hat{\varepsilon }}_{ij}$ and $u_{i}^{p}$ is compatible with $\varepsilon_{ij}^{p}+{\tilde{\varepsilon }}_{ij}$. ${\hat{u}}_{i}$ can be readily determined from an elastic analysis. We want to determine $u_{i}^{p}$. Note the following analysis can be applied in either the loaded or unloaded state and $\varepsilon_{ij}^{p}$ might be different in these two loading conditions.

To determine *u*_*i*_ (or $u_{i}^{p}$), we apply a dummy load $\Gamma_{i}^{*}$ at the location of interest in the direction of the required displacement, as shown in figure 12*b*. We can perform an elastic analysis to determine $\sigma_{ij}^{*}$ that is in equilibrium with $\Gamma_{i}^{*}$ and

6 $$\varepsilon_{ij}^{*}=C_{ijkl}^{-1}\sigma_{kl}^{*}.$$

Note in general we would apply a unit dummy load. If we require an average displacement over a specified area of the surface, we can apply a uniform dummy pressure over this region.

Now applying the principle of virtual work, where ${\tilde{\varepsilon }}_{ij}+\varepsilon_{ij}^{p}$/$u_{i}^{p}$ provides a compatible set and $\Gamma_{i}^{*}$/$\sigma_{ij}^{*}$ an equilibrium set, gives

7 $$\begin{array}{rl} \Gamma_{i}^{*}u_{i}^{\Gamma } & =\int_{V}\sigma_{ij}^{*}(\tilde{\varepsilon_{ij}}+\varepsilon_{ij}^{p})\;\text{d}V \end{array}$$

8 $$\begin{array}{rl} & =\int_{V}\left[\varepsilon_{ij}^{*}C_{ijkl}C_{klmn}^{-1}\rho_{mn}+\varepsilon_{ij}^{p}\sigma_{ij}^{*}\right]\;\text{d}V \end{array}$$

9 $$\begin{array}{rl} & =\int_{V}\varepsilon_{ij}^{*}\rho_{ij}\;\text{d}V+\int_{V}\varepsilon_{ij}^{p}\sigma_{ij}^{*}\;\text{d}V. \end{array}$$

The first term is zero since $\varepsilon_{ij}^{*}$ is a compatible strain field and *ρ*_*ij*_ is in equilibrium with zero applied load.

Now taking the magnitude of the dummy load as unity, equation (7) becomes

10 $$u_{\Gamma }=\int_{V}\varepsilon_{ij}^{p}\sigma_{ij}^{*}\;\text{d}V,$$

where $u_{\Gamma }$ is the component of $u_{i}^{\Gamma }$ in the direction of $\Gamma_{i}^{*}$.

If instead of continuum plasticity, plastic deformation occurs by slip on discrete slip planes, equation (10) becomes

11 $$u_{\Gamma }=\int_{A_{s}}b\sigma_{ij}^{*}n_{i}s_{j}\;\text{d}A$$

note $\sigma_{ij}^{*}n_{i}s_{j}=\tau$ is the resolved shear stress on the slip plane from the application of the dummy load, where *s*_*j*_ is the unit vector in the direction of slip, *b* is the unit of slip, i.e. the magnitude of Burgers vector, *n*_*i*_ is the normal of the slip plane and *A*_*s*_ is the area of the body (total area of slip planes) over which slip has occurred.

Note the displacement $u_{\Gamma }$ is independent of ${\tilde{\varepsilon }}_{ij}$ and *ρ*_*ij*_, which is the strain field generated by the dislocations when plastic deformation occurs by slip. The surface displacement is determined only by the amount of slip (area swept out by the dislocation) and not on the current position of the dislocations, i.e. it is the difference between the current and initial positions that determines the surface placement. Additionally, the elastic deformation ${\tilde{\varepsilon }}_{ij}$ is important to maintain compatibility, but its main purpose is to transmit the effect of the internal inelastic strain to the surface.

For the micropillar considered in §4, application of a unit dummy pressure at the top of a micropillar results in a uniform stress $\sigma_{33}^{*}$ throughout the pillar. The surface displacement, and average strain in the pillar, is then independent of the location of the slipped areas within the pillar.
